# Assessing the influence of diverse phosphorus sources on bacterial communities and the abundance of phosphorus cycle genes in acidic paddy soils

**DOI:** 10.3389/fmicb.2024.1409559

**Published:** 2024-10-10

**Authors:** Affi Jeanne Bongoua-Devisme, Sainte Adelaïde ahya Kouakou, Konan-Kan Hippolyte Kouadio, Bahan Franck Lemonou Michael

**Affiliations:** ^1^Department of Pedology and Agricultural Durable, UFR STRM, FHB University, Abidjan, Côte d'Ivoire; ^2^Center National of Research Agronomic-CNRA, Man, Côte d'Ivoire

**Keywords:** NPK fertilizers, TSP, natural phosphate rock, bacteria phylum, chemical fertilizer

## Abstract

The impact of chemical fertilizers on soil microbial communities is well acknowledged. This study assesses the influence of various phosphorus sources on soil bacterial composition, abundance, and Phosphorus Cycle Gene Abundance. Three phosphorus sources (natural phosphate rock, triple super phosphate (TSP), and chemical fertilizer NPK) were field tested following two rice cultivation cycles. Soil samples were subsequently collected and analyzed for bacterial groups and phosphorus cycle genes. Results indicated that the bacterial community composition remained consistent, comprising five main phyla: Firmicutes, Actinobacteria, Proteobacteria, Halobacterota, and Chloroflexia, regardless of fertilizer type. NPK fertilizer significantly reduced the relative abundance of Chloroflexia by 19% and Firmicutes by 16.4%, while increasing Actinobacteria and Proteobacteria by 27.5 and 58.8%, respectively. TSP fertilizer increased Actinobacteria by 27.1% and Halobacterota by 24.8%, but reduced Chloroflexia by 8.6%, Firmicutes by 12.6%, and Proteobacteria by 0.6%. Phosphate rock application resulted in reductions of Chloroflexia by 27.1%, Halobacterota by 22.9%, and Firmicutes by 6.2%, alongside increases in Actinobacteria by 46.6% and Proteobacteria by 23.8%. Combined application of TSP, NPK, and phosphate rock led to increases in Proteobacteria (24–40%) and Actinobacteria (13–39%), and decreases in Chloroflexia (5.2–22%) and Firmicutes (6–12.3%) compared to the control (T0). While the different phosphorus sources did not alter the composition of phosphorus cycle genes, they did modulate their abundance. NPK fertilizer did not significantly affect ppK genes (57–59%) but reduced gcd (100 to 69%), 3-phytase (74 to 34%), appA (91 to 63%), and phoD (83 to 67%). Phosphate rock reduced appA and gcd by 27 and 15%, respectively, while increasing 3-phytase by 19%. TSP decreased ppK and phoD by 42 and 40%, respectively, and gcd and appA by 34 and 56%, respectively. Combined fertilizers reduced appA (49 to 34%), 3-phytase (10 to 0%), and gcd (27 to 6%), while increasing ppK (72 to 100%). Among tested phosphorus sources, natural phosphate rock was best, causing moderate changes in bacterial composition and phosphorus genes, supporting balanced soil microbial activity. These findings highlight the complex interactions between fertilizers and soil microbial communities, underscoring the need for tailored fertilization strategies to maintain soil health and optimize agricultural productivity.

## Introduction

Phosphorus (P) is essential for plant growth, but its accessibility in acidic tropical soils is often limited (Kpan et al., 2023). To overcome this challenge, chemical fertilizers, whether organic or inorganic, are widely employed to enhance phosphorus availability and boost crop yields (Sahrawat et al., 2001; Koné et al., 2023; Kpan et al., 2023). The application of mineral and organic fertilizers has proven effective in improving soil fertility and crop productivity, serving as primary nutrient sources for arable soils (Wang et al., 2017; Wu et al., 2021; Kpan et al., 2023). For instance, Islam et al. (2015) reveal the positive impact of Nitrogen (N) fertilizer on wheat grain yield, while Das et al. (2023) emphasize the stabilizing effect of long-term manure application on soil organic matter and overall soil health. Nitrogen treatments were found to influence soil pH, cation exchange capacity (CEC), and phosphorus levels (Setu, 2022; Wang et al., 2023; Zeng et al., 2024).

Microbes in agricultural soil play pivotal roles in nutrient cycling, participating in processes such as organic matter decomposition and the biogeochemical cycling of elements (C, N, P, S) (Morris and Blackwood, 2015). They contribute essential nutrients for crop growth, and alterations in soil nutrient levels post-fertilization significantly influence microbial biomass, composition, and diversity. The sensitivity of soil microbial biomass and diversity to variations in soil nutrients, pH, and organic matter content has been well-documented (Wang et al., 2017). Furthermore, the growth and activities of bacteria and fungi exhibit adaptability in response to crop yield and the chemical, physical, and biological properties of the soil (Kou et al., 2023; Zeng et al., 2024).

Several studies have documented shifts in soil microbial communities following fertilizer application (Sivojiene et al., 2021; Wang et al., 2022). For example, Sivojiene et al. (2021) observed an increase in microbial biomass and diversity with organic fertilizations, while Wang et al. (2022) noted the impact of different fertilization treatments on the bacterial community. Ren et al. (2018) and (2020) highlighted that adequate nitrogen and carbon from organic fertilizer supplementation promote soil microbial growth, but excess phosphorus from fertilization reduces microbial community diversity (Liu et al., 2022). The application of fertilizers, particularly chemical ones, can lead to various adverse effects on soil properties and indirectly impact its microbial ecosystem (Cheng et al., 2020). Continuous application of chemical fertilizers can cause soil acidification, negatively affecting soil structure and fertility (Pahalvi et al., 2021). Imbalanced use of chemical fertilizers can alter soil pH, increase pest attacks, and exacerbate acidification (Krasilnikov et al., 2022). Consequently, acidification can lead to the loss of essential nutrients, decreased soil pH, and reduced availability of micronutrients, ultimately resulting in poorer crop performance and soil degradation (Ozlu and Kumar, 2018).

Fertilizers can also alter the composition and functioning of microbial communities (Kai et al., 2020). They affect soil microorganisms indirectly by changing soil properties or directly through the addition of nutrients (Pan et al., 2020; Yan et al., 2021). Research has shown that prolonged use of chemical fertilizers can significantly reduce soil bacterial diversity, primarily due to a decrease in soil pH levels (Xu et al., 2020; Yang et al., 2020). While some research suggests that organic fertilizer application can increase soil microbial diversity compared to chemical fertilizers (Sun et al., 2015; Kai et al., 2020), other studies indicate the opposite (Hu et al., 2018; Xu et al., 2020; Yang et al., 2020). According to Zhang et al. (2022), while moderate fertilizer use may enhance microbial activity and diversity, excessive application can lead to a decline in beneficial microbes and an increase in pathogenic species. Additionally, the use of combined inorganic and organic fertilization in rice field soils across South China was found to change the abundance of soil microbial communities (Liu et al., 2020). To mitigate the negative impacts of intensive fertilizer use, there is a pressing need for comprehensive and sustainable soil management practices. This has led to a growing interest in eco-friendly and cost-effective technologies, including the utilization of phosphate rock (PR), a low-cost natural resource recognized as a sustainable alternative for agriculture (Vassilev et al., 2001; Reddy et al., 2002).

In this context, our study aims to elucidate the impact of three phosphorus sources—natural phosphorus rock of Morocco (PRM), Triple Super Phosphate (TSP), and NPK 15/15/15—on the composition of soil bacterial groups and the genes involved in the phosphorus cycle.

## Materials and methods

### Study sites and soil sampling

Our research was conducted in the western region of Ivory Coast from 2019 to 2021, specifically in a rice field situated at the National Center of Agricultural Research (CNRA) station (7° 18′57” N; 7° 27′19” W). The experiment was conducted during the main cropping season, characterized by a mono-modal rainfall pattern. The area receives an average annual rainfall of 1771 mm (Koné et al., 2010). The rainy season extends from February to October, with peak rainfall occurring between March and June. The climate is warm and humid, with mean annual maximum and minimum temperatures of 32°C and 17°C, respectively, and an average relative humidity of 79%.

Before initiating the experiments, soil samples were systematically collected at a depth of 0–20 cm from various locations within the plot to ensure comprehensive coverage and representativeness. These individual subsamples were then combined into a composite sample, which was subsequently sieved (2 mm) and divided into two parts. The first part underwent thorough physico-chemical analysis, while the second part was stored at −4°C for microbiological analysis. The soil’s characterization before experimentation is presented in Table 1.

**Table 1:** Physico-chemical and microbiology characteristics of the paddy soils at 0–20 cm depth before experimentation.

Parameters	Upland	Lowland
Clay (%)	29	6
Silt (%)	16	13
Sand (%)	55	81
pH water	5,2	5,6
pH KCl	3,6	4,3
Assimilable P (g kg^−1^ dry soil)	5	2,1
Organic C (g kg^−1^ sol sec)	143	69
Total N (g kg^−1^ sol sec)	13	7
Organic Matter (g kg^−1^ sol sec)	246	120
C/N	11	9,8
K^+^ (g kg^−1^ sol sec)	0,96	0,22
Na^+^ (mmol+.kg^−1^)	0,08	0,03
Ca^++^ (mmol+.kg^−1^)	4,96	2,47
Mg^++^ (mmol+.kg^−1^)	2,4	0,78
CEC (mmol+.kg^−1^)	8,5	3,4
S/T (%)	15,66	10,27
TB (10^5^ /g dry soil)	833	23,000
PSB (10^5^ /g dry soil)	5	1

TB, total bacterial; PSB, phosphate solubilizing bacterial.

#### Plant material

Two rice varieties, WITA 9 and IDSA 10, were thoughtfully selected from the National Center of Agricultural Research (CNRA) of Ivory Coast, taking into consideration the specific ecologies of the plot. The rice variety WITA 9 is also called Nimba. It was developed in 1984 by the International Institute of Tropical Agriculture (IIAT) by mixing the variety HI 2042-178-1 and the variety CT19 is an improved variety which was chosen mainly for its short cycle (90 days). It’s average yield of 6 t ha^−1^ and its potential yield of 10 t ha^−1^. The seed of WITA 9 provided in National Center of Agricultural Research (CNRA) in Man. The rice variety IDSA 10, also known as Fafa, was provided by the National Center for Agronomic Research (CNRA) in Man. Resulting from a cross between IRAT 112 and Iguape Cateto, it is suited to uplands and slopes and has a short growth cycle of 105 days. Its potential grain yield is 4.8 t/ha. However, in agricultural practice in Côte d’Ivoire, the average harvest is 2.5 t/ha, which can vary depending on the agroecology. This variety is widely adopted in the country.

#### Phosphorus sources material

Three different phosphorus sources were employed in this study. The first is a natural phosphorus rock, specifically Morocco phosphate rock (PR), boasting a P_2_O_5_ content of 30% with a solubility of 3% in water (Table 2). The second is a chemical phosphorus fertilizer, Triple Superphosphate (TSP), also containing 45% P_2_O_5_. The third phosphorus source is NPK fertilizer (15/15/15) was incorporated into the study to ensure a recommended dose of NPK for rice and was applied at a dose of 200 kg NPK ha^−1^. Both fertilizers (Morocco phosphate rock (PR) and Triple Superphosphate (TSP)) were generously supplied by the Office Cherifien of Phosphate (OCP) and was applied at a dose of 90 kg P_2_O_5_ ha^−1^ or 300 kg TSP or PR per hectare. Another additional nutrient, such as a nitrogen fertilizer in the form of Urea (46% N) was employed at a dose of 100 kg Urea ha^−1^. The Table 3 gives the composition of the different treatments applied.

**Table 2:** Elemental chemical composition of Moroccan phosphate rock (MPR) analyzed in 100 g MPR samples.

Chemical composition	P_2_O_5_	CO_2_	SO_3_	CaO	MgO	Fe_2_O_3_	Al_2_O_3_	F_2_O	H_2_O	SiO_2_
Content (%)	30	6.44	1.29	49.55	1.16	0.20	0.4	2.21	2.11	6.64

**Table 3:** Treatments composition and values of fertilizers and nutrients applied on plots.

Treatments	Quantities of fertilizer applied (kg ha^−1^)				Quantity of nutrients added to each treatment (kg ha^−1^)		
	PRM	TSP	NPK	Urea	N	P	K
T0a (soil potential)	0	0	0	0	0	0	0
T0 (NPK, reference control)	0	0	200	100	76	13.2	24.9
T1 (100% PRM)	300	0	200	100	76	52.8	24.9
T2 (90% PRM +10%TSP)	270	30	200	100	76	52.8	24.9
T3 (80% PRM +20%TSP)	240	60	200	100	76	52.8	24.9
T4 (40% PRM +60%TSP)	120	180	200	100	76	52.8	24.9
T5 (20% PRM +80%TSP)	60	240	200	100	76	52.8	24.9
T6 (100 %TSP)	0	300	200	100	76	52.8	24.9

#### Trial design

The experimental setup used was a randomized complete block design (RCBD), with a single application of treatments in the first cycle. The experiment was conducted on a 500 m^2^ plot using a randomized complete block design, with 100 m^2^ blocks serving as replications. Five replication was considered in each Block design. These blocks were then subdivided into 8 microplots of 25 m^2^ each, with each microplot representing a distinct treatment. Different treatments based of different phosphorus sources (PR, TSP, NPK) were applied, at a dose of 90 kg P_2_O_5_ ha^−1^ or 300 kg TSP or PR per hectare, only at the beginning of the first cycle in the plots, excluding the control treatment (T0). Additionally, 100 kg ha^−1^ of 46% Urea was applied, with 50 kg ha^−1^ at the tillering stage and 50 kg ha^−1^ at the at panicle stage. For upland plots, after applied the treatment, seeds were directly sown at a rate of four seeds per hole. After germination, thinning was carried out to leave two plants per hole before tillering. To avoid competition between the rice and weeds, manual weeding was performed as needed. No insecticides or fungicides were applied to the plots.

For lowland plots, two days before sowing, the 15 day-old rice plants, NPK 15-15-15 (200 kg ha^−1^) and the different treatments were applied, as background fertilizer for each plot except for the absolute control. The transplanting was carried out at a rate of 02 plants per pocket.

Microbiological characterization soil samples were collected by treatment at the end of the second cropping cycle.

#### Quantification and analysis of microbial diversity in rice plot soils

The analysis of the bacterial communities present in soils was carried out using molecular biology based on the extraction of total DNA from the indigenous bacterial at ecology laboratory of the Institute of research of development in agroenvironment (IRDA) in Quebec, Canada (Quebec, QC Canada).

#### Extraction, amplification, and sequencing of total bacterial DNA from soils

DNA from soil samples was extracted from 0.5 g of soil using the FastDNA Spin kit for Soil (MP Biomedicals, Solon, OH, United States), according to manufacturer’s instructions at the Laboratory of Microbial Ecology of the IRDA (Québec, QC Canada) with three replicates per sample. Extracted DNA was stored at −20°C for amplification and sequencing.

Bacterial DNA amplification focused on the V4 regions of prokaryotic 16S rRNA, employing primer sequences specific to the V4 regions of the SSU rRNA gene (515F and 806R) following the methods outlined by Apprill et al. (2015) and Parada et al. (2016) (Table 4). The chosen approach involved a dual-index, two-step PCR designed for Illumina MiSeq high-throughput sequencing. Consequently, DNA was amplified with primers 515F/806RB and sequenced to evaluate amplification bias and sequencing error rate as described (Tekeu et al., 2023). Paired-end sequencing (2 × 300 base pairs) occurred on Illumina MiSeq at the genomic analysis platform at the Institute of Research and Development in Agroenvironment (IRDA) in Quebec, Canada (Quebec, QC Canada).

**Table 4:** List of the primers that were used in this study/primers used in this study.

Name of primers	Sequences (5′ → 3′)	References
515F (modified)	GTGYCAGCMGCCGCGGTAA	Parada et al. (2016)^20^
926R	CCGYCAAATTYMTTTRAGTTT	Parada et al. (2016)^20^
806R (modified)	GGACTACNVGGGTWTTCTAAT	Apprill et al. (2015)^19^

#### Quantitative PCR

The qPCR system utilized primers eub338/eub518 (Fierer et al., 2005) to detect total bacteria. Detection was conducted in duplicate on a CFX96 instrument (Biorad, Hercules, CA, United States) using SYBR green qPCR mix (Qiagen, Toronto, ON, Canada), following the procedure outlined in Tekeu et al. (2023). The detection system was designed within a detection range spanning 4 LOG. This method served to quantify the relative abundance of bacteria in the soil samples, expressed as the number of targeted sequences (Amplified Units) per gram of dry soil (AU g^−1^ dry soil). Subsequently, these values underwent normalization, and a log transformation was applied.

#### Predictive functions of phosphate-solubilizing bacteria

Phosphate-solubilizing bacteria are characterized by several biochemical activities, including acid production and enzyme. These functions are determined from taxonomic data obtained by analyzing the diversity of prokaryotes. As part of this study, several genes involved in phosphate solubilization by bacteria or that could serve as biomarkers were identified and mentioned as method described by Wan et al. (2020) and Wu et al. (2022).

#### Bioinformatics and biostatistics processing

Sequence analysis and grouping into sequence taxonomic units was performed on IRDA’s LEM bioinformatics platform and involved various processing strategies Qiime2 (Bolyen et al., 2019) and R (R Core Team project, 2014), including quality validation steps, reference bases and indices for measuring microbial richness, and comparative measures of microbial diversity. These were grouped into OTUs based on 97% sequence similarity. Taxon assignment was performed using the SILVA version 138 reference database (Quast et al., 2012), which was also used for the analysis of bacterial diversity. For the analysis of predictive functions related to phosphate solubilization, the data obtained from the taxonomic analysis were processed using the Picrust2 approach (Douglas et al., 2019) and 5 marker genes related to this functionality were filtered.

#### Statistical analysis

Statistical models were developed using the lm function from the agricolae package in R software version 4.3.2 (R Core Team, 2023), with its RStudio interface. The data are presented as mean ± standard deviation. Statistical differences were considered significant for *p* < 0.05. The significant difference between treatments was analyzed by Newman–Keuls test in one-way ANOVA and multiple comparisons of means (*p* < 0.05) were conducted using the Newman–Keuls test to identify homogenous groups at the 5% probability threshold.

## Results and discussion

### Results

#### Treatment effect on the relative abundance of bacterial community composition

**Figure 1:**
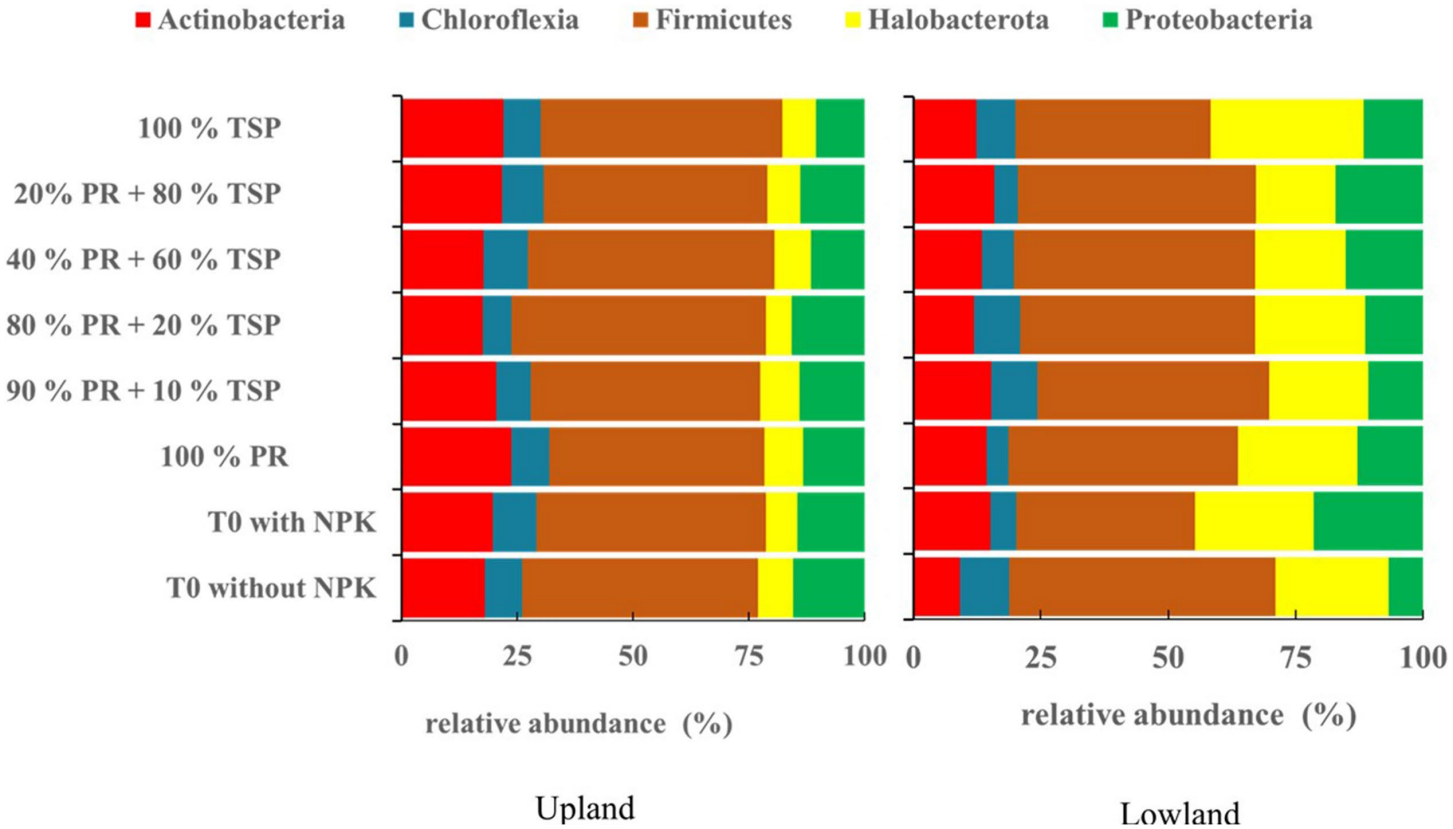
The relative abundance (%) of bacterial community composition among the primary prokaryotic groups detected in upland and lowland habitats (groups greater than 1%) under different treatments.

The addition of different phosphate amendment does not alter the soil indigenous bacterial community composition in the two rice plots studied, with the presence of five (5) phyla Firmicutes, Actinobacteria, Proteobacteria, Halobacterota, and Chloroflexia (Figure 1), regardless of the treatments applied and the ecological characteristics of the plots (upland/lowland).

The Firmicutes phylum (35 to 53.25%) is the most dominant, followed by the Halobacterota phylum (6.7 to 30%), Actinobacteria phylum (9 to 23.8%), and Proteobacteria phylum (5 to 21%) in each phylum (Figure 1). While, the Chloroflexia phylum (4 to 9.58%) appears least abundant phylum in the both rice plots (Figure 1).

**Figure 2:**
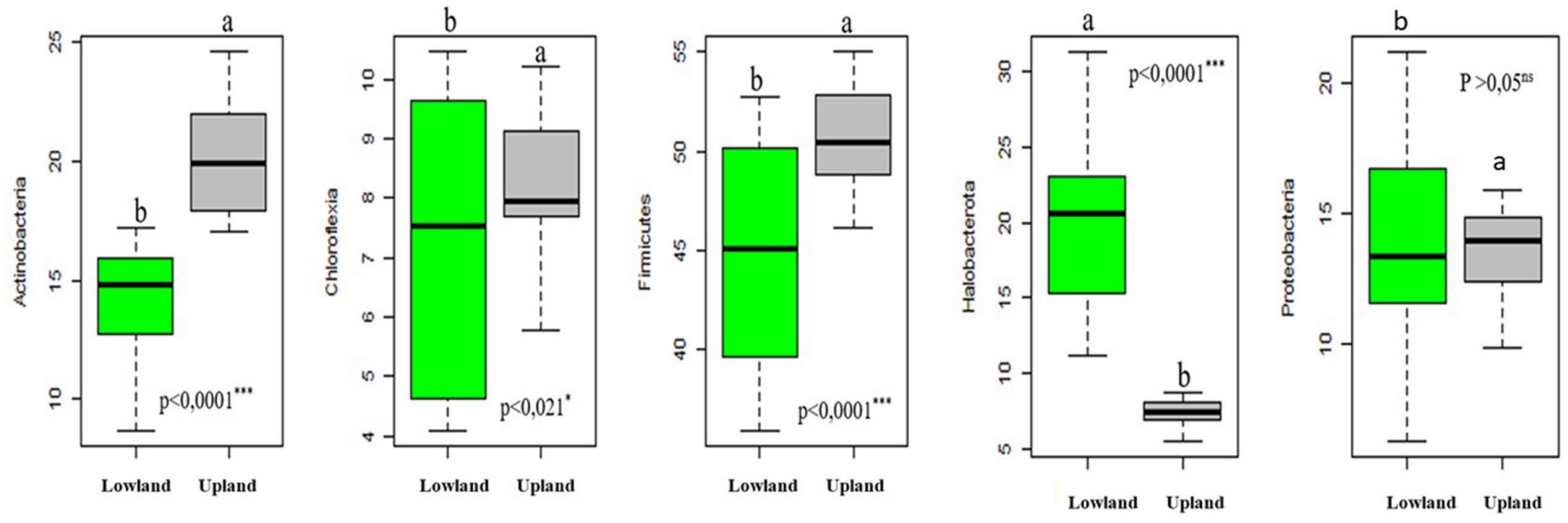
Boxplot showing the relative abundance bacterial groups (phyla) for two-habitats plots studied (upland and lowland) (groups greater than 1%). The thick horizontal line represents the median of the distribution. Different letters indicate differences among medians according to the Tukey test (*p* < 0.001).

Our results demonstrate that the relative abundance of different phyla varied between the two ecological plot types studied, irrespective of the treatment applied. Specifically, Actinobacteria, Chloroflexia, Firmicutes and Proteobacteria phlyla exhibited significantly higher relative abundances in upland plots compared to lowland plots (Figure 2 and Table 5). Conversely, for Halobacterota phylum, their relative abundance was higher in lowland plots than in upland plots (Figure 2 and Table 5).

**Table 5:** Plot habitat influence on bacterial community’s (phylum group).

Habitat	Phylum group	*R* ^2^	*F* values	Pr (>*F*)	*β*	*α*
Lowland	Actinobacteria	0.0739	3.0338	0.0896	0.2764	12.822
	Chloroflexia	0.0074	0.2828	0.598	−0.084	7.5911
	Firmicutes	0.15^*^	6.6976	0.0136	−0.9348	49.0532
	Halobacterota	0.0439	1.7429	0.195	0.5018	17.748
	Proteobacteria	0.0188	0.7281	0.399	0.2406	12.7857
Upland	Actinobacteria	0.0588	2.3741	0.132	0.2327	19.0677
	Chloroflexia	0.0054	0.2082	0.651	0.0346	7.9914
	Firmicutes	0.0767	3.1564	0.0836	0.3036	49.3794
	Halobacterota	0.0229	0.8887	0.352	−0.0573	7.6654
	Proteobacteria	0.445^***^	30.4591	*p* < 0.0001	−0.5136	15.8961

*statistically significant at the 0.05 level (*p* < 0.05), **statistically significant at the 0.01 level (*p* < 0.01), and ***statistically significant at the 0.001 level (*p* < 0.001).

**Figure 3:**
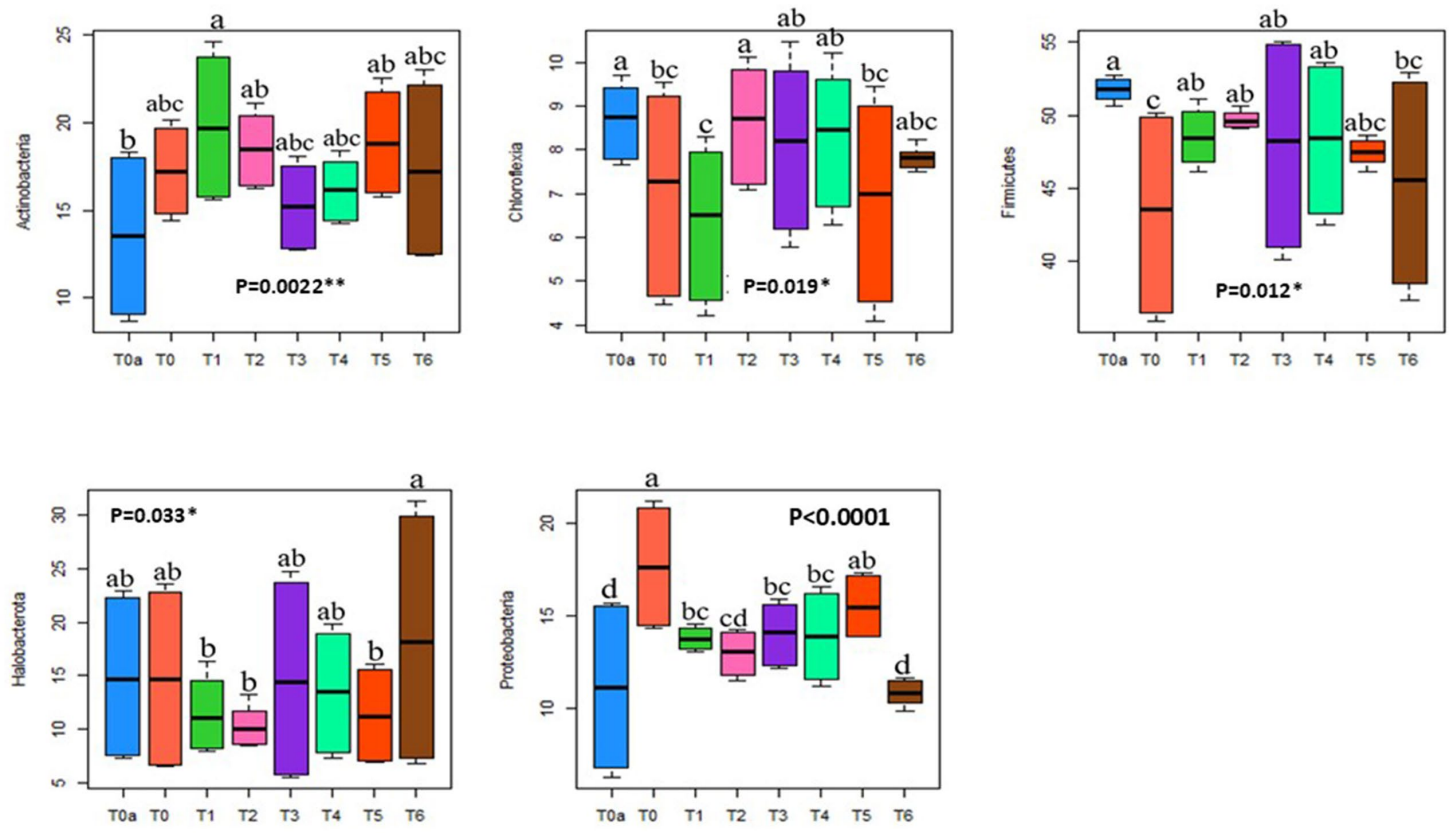
Boxplot showing the relative abundance of bacterial phyla among the primary prokaryotic groups detected (greater than 1%) under different treatments. The thick horizontal line represents the median abundance of each phylum within each treatment. Blue indicates T0a (soil potential without NPK, PRM, and TSP), orange indicates T0 (reference control with NPK), dark green indicates T1 (100% PRM), pink indicates T2 (90% PRM + 10% TSP), violet indicates T3 (80% PRM + 20% TSP), light green indicates T4 (40% PRM + 60% TSP), red indicates T5 (20% PRM + 80% TSP), and brown indicates T6 (0% PRM + 100% TSP).

Indeed, the addition of soluble NPK fertilizer decreases the relative abundance of the Chloroflexia phylum from 9% under T0a to 5% under T0 (with NPK), and of the Firmicutes phylum from 52.3% under T0a to 36.7% under T0 (Figure 3), regardless of plot habitat. As for the Actinobacteria and Proteobacteria phyla, there is a significant notable increase in relative abundance, respectively, from 9% under T0a to 19.6% under T0, and from 6% under T0a without NPK to 20.9% under T0 (Figure 3). Additionally, it is observed that the addition of NPK does not affect the relative abundance of Halobacterota compared to the control T0a (Figure 3).

Our findings demonstrate that amending the plot with phosphate rock from Morocco (100% PRM, T1) significantly alters the relative abundance of the five detected phyla compared to the unamended plot (T0a). The presence of phosphate rock (PRM) significantly reduces the relative abundance of the Chloroflexia (from 8.7 to 6.31%), Halobacterota (from 14.9 to 11.5%), and Firmicutes (from 51.8 to 48.6%) phyla, while increasing the relative abundance of Actinobacteria (from13.5 to 19.8%) and Proteobacteria (from 11.1 to 13.8%), compared to the control treatment (T0a) (Figure 3).

On the other hand, when plots were amended with a combination of phosphate rock and triple superphosphate (TSP), particularly in treatments T3 (80% PRM + 20% TSP) and T4 (40% PRM + 60% TSP), resulted in a reduction in the relative abundance of Chloroflexia (from 8.65 to 8%), and Firmicutes (from 51.8 to 47.92%), alongside an elevation in the relative abundance of Proteobacteria (from 11.1 to 14%) and Actinobacteria (from 13.5 to 16.2%), compared to the untreated plot (T0a) (Figure 3). Additionally, the relative abundance of Halobacterota remained relatively constant at 14.93 to 13.5% compared to the unamended plot (T0a).

Furthermore, when plots received only Triple Super Phosphate (TSP) as the phosphate amendment, compared to the control treatment (T0a), significant modifications were observed in the relative abundance of the five detected phyla. The presence of TSP alone decreased the relative abundance of Chloroflexia (from 8.65 to 7.8%) and Firmicutes (from 51.8 to 45.4%), while increasing the relative abundance of Actinobacteria (from 13.5 to 17.4%), as well as Halobacterota (from 14.93 to 18.6%), compared to the unamended plot (T0a) (Figure 3). There was no significant effect on the relative abundance of Proteobacteria remained relatively constant at 11.13 to 10.8% compared to the unamended plot (T0a).

#### Treatment effect on phosphorus cycle genes

Whatever the habitat of the rice plots (upland/lowland) and treatment applied, five (5) phosphorus cycle genes were identified in the soils. These were (Table 6):

- Enzymes involved in inorganic phosphate solubilization (gcd [EC:1.1.5.2] quinoprotein glucose dehydrogenase and ppk [EC:2.7.4.1] polyphosphate kinase).
- Enzymes involved in organic phosphate mineralization (3-phytase [EC:3.1.3.8], appA [EC:3.1.3.26 3.1.3.2] 4-phytase/acid phosphatase and phoD [EC:3.1.3.1] alkaline phosphatase D).

**Table 6:** Phosphorus cycle genes identified on the plots studied.

Metabolic pathway	Genes	Enzyme	KEGG ID (Ko)
inorganic phosphates Solubilisation	gcd [EC:1.1.5.2]	Quinoprotein Glucose Dehydrogenase	K00117
	ppk [EC:2.7.4.1]	Polyphosphate kinase	K00937
organic phosphate Mineralization	3-phytase [EC:3.1.3.8]	3-phytase	K01083
	appA [EC:3.1.3.26 3.1.3.2]	4-phytase/acid phosphatase	K01093
	phoD [EC:3.1.3.1]	alkaline phosphatase D	K01113

KEGG, Kyoto encyclopedia of genes and genomes.

**Figure 4:**
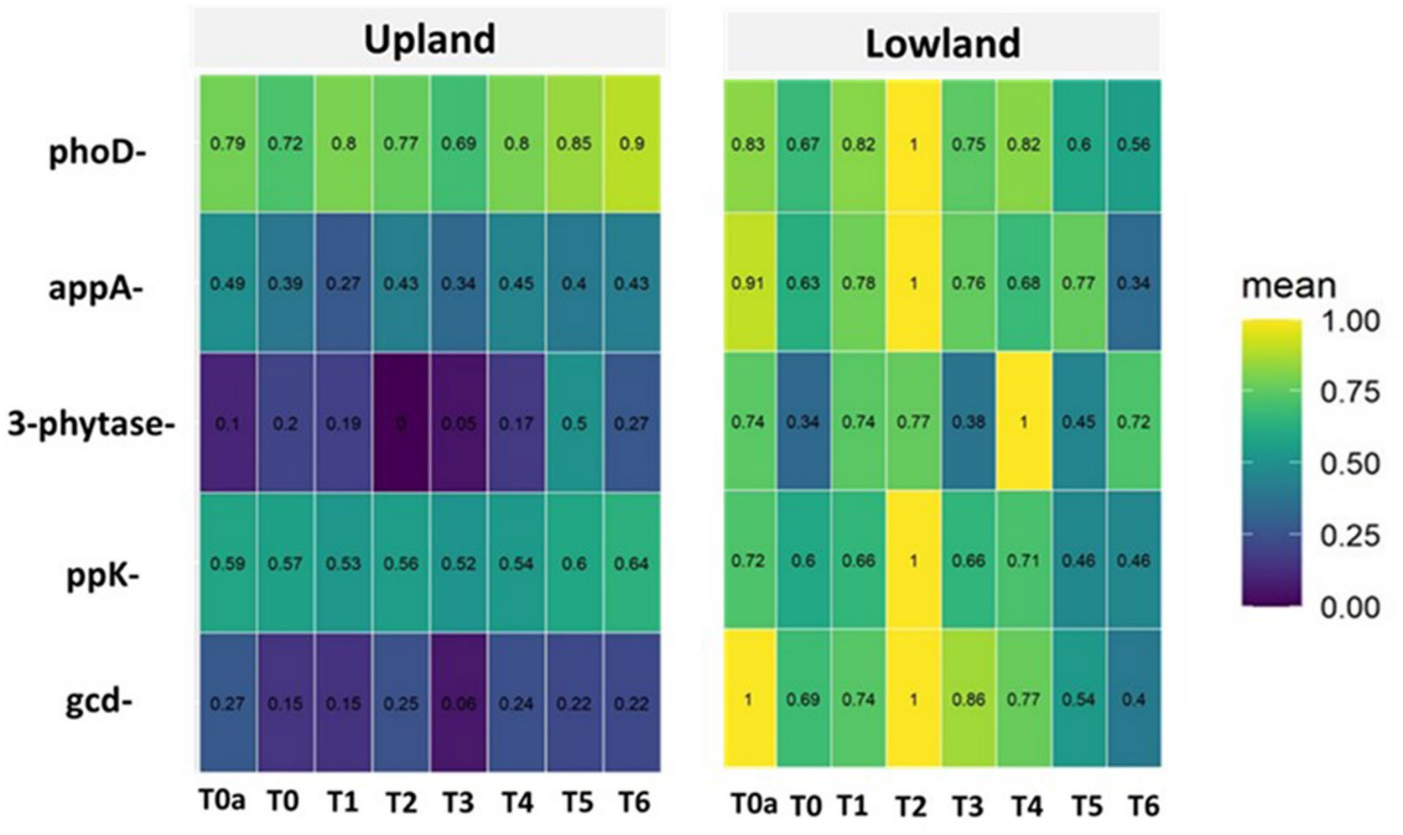
Abundance of phosphorus cycle genes identified in upland and lowland habitat. Treatments: T0a (absolute control without NPK), T0 (Control with NPK), T1 (100% RP); T2 (90% RP + 10% TSP), T3 (80% RP + 20% TSP), T4 (60% RP + 40% TSP), T5 (20% RP + 80% TSP), T6 (100% TSP).

In our study, among the five phosphorus cycle genes identified in the soil plots, the phoD and ppk genes were found to have the highest relative abundance in upland soils, regardless of the applied treatment. However, in lowland soils, the phoD, ppk, and gcd genes were identified as having the highest relative abundance (Figure 4).

The application of treatments has influenced the abundance of phosphorus cycle genes identified.

Indeed, when compared to soil potential (T0a, without NPK, control), it is evident that the addition of soluble NPK fertilizer decreases in upland, the relative abundance of appA gene (from 49% under T0a to 39%) under T0 (with NPK), and of gcd gene (from 27% under T0a to 15%) under T0 (Figure 4). As for 3-phytase gene, there is a significant notable increase in relative abundance, from 10% under T0a to 20% under T0 (Figure 3). Additionally, it is observed that the addition of NPK does not affect the relative abundance of phoD (79 to 72%) and ppK (59 to 57%) genes compared to the control T0a (Figure 4). In lowland, there is a significant notable decrease in relative abundance of gcd (100 to 69%), 3-phytase (74 to 34%), appA (91 to 63%) and phoD (83 to 67%).

Our findings demonstrate that amending the plot with phosphate rock from Morocco (100% PRM, T1) significantly reduces the abundance of appA (49 to 27%) and gcd (27 to 15%) genes, compared to the unamended plot (T0a), in upland plot, while increasing the relative abundance of 3-phytase (10 to 19%). Additionally, it is observed that the presence of PRM does not affect ppk gene (59 to 53%), and phoD (79 to 80%), compared to the control treatment (T0a) (Figure 4). In lowland, the presence of PRM significantly reduces the abundance of appA (91 to 78%), ppk (72 to 66%), and gcd (100 to 74%) genes, compared to the unamended plot (T0a). There was no significant effect of PRM on the abundance of phoD and 3-phytase genes remained relatively constant, respectively, at 83 to 82% and at 74 to 74%, compared to the unamended plot (T0a).

On the other hand, when plots were amended with a combination of phosphate rock and triple superphosphate (TSP), particularly in treatments T3 (80% PRM + 20% TSP) and T4 (40% PRM + 60% TSP), resulted in upland a reduction in the abundance of appA (49to 34%), 3-phytase (10 to 0%), gcd (27 to 6%), alongside there was no significant effect of PRM on the abundance of phoD and ppk genes compared to the unamended plot (T0a) (Figure 4). In lowland, the combination of phosphate rock and triple superphosphate (TSP) significantly reduces the abundance of appA (91 to 68%), phoD (83 to 75%), and gcd (100 to 77%) genes, compared to the unamended plot (T0a), while increasing the relative abundance of 3-phytase (74 to 100%) and ppK (72 to 100%) genes (Figure 4).

Furthermore, when plots received only Triple Super Phosphate (TSP) as the phosphate amendment, compared to the control treatment (T0a), significant modifications were observed in the abundance of phosphorus genes. In lowland plot, the presence of TSP alone decreased the abundance of ppk (72 to 42%), gcd (100 to 40%), appA (91 to 34%), and phoD (83 to 56%), genes, while there was no significant effect on the abundance of 3-phytase (74 to 72%). In upland plot, the presence of TSP alone increased the abundance of ppk (59 to 64%), phoD (79 to 90%) and 3-phytase (10 to 27%) genes, while there was no significant effect on the abundance of gcd (27 to 22%) and appA (49 to 43%) genes (Figure 4).

## Discussion

In agricultural soil, in order to mitigate the limited availability of phosphorus nutrients, agriculturists commonly resort to artificial supplementation through both inorganic and organic fertilization methods. Soil fertilization has been shown to improve bacterial diversity and abundance in the soil (Sun et al., 2015).

### Impact of plot habitat on composition and abundance bacterial communities

The investigation carried out on rice field soils from both upland and lowland plots at the Man Research Station revealed the presence of five (05) phyla bacterial (Firmicutes, Actinobacteria, Proteobacteria, Halobacterota, Chloroflexi) in plots of both ecologies, indicating stability in bacterial communities composition regardless ecological differences. However, variations in the relative abundance of phyla were observed depending on the plot habitat, likely attributable to divergent agricultural practices employed in these two ecological settings. Numerous studies indicate that various soil management practices strongly affect the soil microbiota by enhancing bacterial community structure and simultaneously improving soil health (Khmelevtsova et al., 2022; Su et al., 2022; Singh et al., 2023; Górska et al., 2024). In fact, in lowland areas, the cultivation technique is based on incorporating organic residues into the soil before transplanting, while on the upland, it involves burning and then direct seeding. This finding is consistent with previous studies by of Szoboszlay et al. (2017) and Li et al. (2018), which highlight the adverse effects of certain agricultural practices, particularly plowing, on fungi presence by disrupting hyphae. Consequently, numerous prior investigations have underscored the profound impact of agricultural practices on microbial communities (Geisler, 2009), with evidence indicating influence on the abundance of denitrifying bacteria groups (Assémien et al., 2017). This study suggests that the composition of bacterial communities in soils remains relatively stable across different ecologies, yet modifications in the relative abundance of phyla occur, as proposed in the works of Vian et al. (2009).

### Effect of diverse phosphorus sources on the composition and abundance soil bacterial groups

The study investigated how different phosphorus sources, such as natural phosphate rock from Morocco (PRM) and chemical fertilizers (Triple super phosphate (TSP) and NPK fertilizers), impact soil bacterial groups. Intriguingly, despite the various treatments applied, the composition of bacterial communities remained unaltered, comprising five main phyla: Firmicutes, Actinobacteria, Proteobacteria, Halobacterota, and Chloroflexia.

Our findings revealed that the application of soluble NPK alone did not alter the composition of bacterial indigenous groups in the studied soils. Regardless of the treatments, the presence of all five phyla remained unchanged. However, compared to the control treatment (T0a, without NPK), the relative abundance of certain phyla in the soil varied in response to NPK application. Specifically, it reduced the relative abundance of Chloroflexia by 19% and Firmicutes by 16.4%, while increasing the relative abundance of Actinobacteria and Proteobacteria by 27.5 and 58.8%, respectively. This finding contrasts with the study by Ma et al. (2018), which showed low abundance levels of Proteobacteria under NPK treatment. Notably, the application of soluble NPK fertilizer alone seemed to affect the relative abundance of soil bacterial groups but not their diversity, as demonstrated by Zhou et al. (2021), which observed a decrease in certain soil bacterial classes under both low and high NPK application. Our results align with several previous studies that reported an increase in the abundance and activity of ammonium-oxidizing bacteria (AOB) in response to NPK fertilizer addition (Simonin et al., 2015; Assémien et al., 2017). According to Rao et al. (2021), this is because NPK fertilization only affects the quantity of soil organic matter rather than its diversity, resulting in minimal impact on the functional structure of the soil.

Similarly, the application of phosphate rock from Morocco (100% PRM, T1) alone resulted in variable changes in the relative abundance of phyla. Specifically, reductions of 27.1% for Chloroflexia, 22.9% for Halobacterota, and 6.2% for Firmicutes were observed, alongside increases of 46.6% for Actinobacteria and 23.8% for Proteobacteria. This highlights the differing impacts of natural phosphate rock on soil bacterial community distribution. Previous studies have shown that the application of rock phosphate improves the phosphorus (P) content in soil, causing significant changes in the soil microorganism community (He et al., 1997; Khoshru et al., 2023; Muhammad Bello et al., 2023). This enhancement in phosphorus availability can lead to shifts in microbial populations, fostering a more diverse and active soil microbiome, as demonstrated by Silva et al. (2017). Furthermore, the variation in the relative abundances of different bacterial groups identified could be linked to soil phosphorus forms, as suggested by Wu et al. (2022).

The use of chemical triple superphosphate (TSP) fertilizer led to increases of 27.1% for Actinobacteria and 24.8% for Halobacterota, while reducing the relative abundance of Chloroflexia by 8.6%, Firmicutes by 12.6%, and Proteobacteria by 0.6%. These findings align with Trabelsi et al. (2017), who observed an increase in Actinobacteria abundance in TSP-treated soil. In contrast, previous studies like Silva et al. (2017) noted a decline in Proteobacteria and Chloroflexi under chemical TSP fertilization compared to untreated soil. Additionally, Peine et al. (2019) demonstrated that TSP application alone boosted microbial biomass and subsequent phosphorus uptake in the soil compared to unfertilized controls.

Additionally, our results revealed that the combination of chemical fertilizers (TSP, NPK) and natural phosphate rock resulted in an increase in the abundance of Proteobacteria (24 to 40%) and Actinobacteria (13 to 39%), and a decrease in the relative abundance of Chloroflexia (5.2 to 22%) and Firmicutes (6 to 12.3%) compared to the control treatment (T0). This study demonstrates that the combination of phosphate rock and chemical fertilizers, such as triple superphosphate modifies bacterial abundance, either increasing or decreasing it (Silva et al., 2017). Similar effect has been observed by Kou et al. (2023); which noted significantly changed the microbial community structure, increasing microbial activity (microbial biomass and soil respiration) when N was combined to P fertilizers. Our results indicate that when chemical fertilizers are combined with phosphate rock, their effects are mitigated, particularly on Firmicutes and Halobacterota. This result does not differ from those of Lori et al. (2023) and Wang et al. (2018), who reported that bacterial α-diversity was unaffected by fertilization intensity, while their community structure changed consistently.

These findings illuminate the intricate relationship between soluble NPK, TSP fertilizers, and natural phosphate rock and their impacts on the relative abundance of bacterial groups in soils. They highlight that each of these phosphorus sources exerts distinct effects on soil bacterial communities. Furthermore, the response of these bacterial groups to phosphate amendments appears to be intricately linked to their inherent characteristics and resilience, as suggested by Kumar et al. (2013). This study affirmed that in soil, the resistance and resilience of microbial populations vary according to their characteristics and nature. For instance, fungi and actinomycetes exhibit greater resistance and resilience compared to other groups of organisms.

These findings imply that soil bacteria possess varying degrees of adaptability and sensitivity to changes induced by different phosphorus sources. This complexity underscores the intricate nature of soil microbial ecology and emphasizes the importance of gaining a nuanced understanding of how soil bacteria interact with environmental factors, including phosphorus availability and soil pH, to uphold ecosystem balance and function.

### Effect of phosphate amendments on the abundance of phosphorus cycle genes in soils

The analysis of phosphorus cycle genes involved in phosphate rock dissolution within rice field plots revealed the presence of five key phosphorus cycle genes (phoD, appA, ppk, gcd, 3-phytase), regardless of plot habitat. Our findings indicate that these diverse P-cycle genes are exclusively attributed to bacterial populations. This corroborates the observations of Siles et al. (2022), who highlight that 3-phytase and phoD genes are present in archaea, fungi, and bacteria, while the appA gene is solely found in bacteria. Similarly, Li et al. (2018) and Enebe and Babalola (2021), assert that gcd and ppk genes originate from bacteria. Specifically, the ppk gene serves as the primary enzyme catalyzing polyphosphate synthesis in bacteria and is essential for bacterial mobility (Zhu et al., 2005), while the gcd gene plays a pivotal role in microbial phosphorus metabolism (Li et al., 2018). Our results showed the abundance of P-cycle gene is higher in lowland than upland habitat, probably due to soil environment or characteristic as suggested by Li et al. (2022).

Inputs from various phosphorus sources do not exert influence on the composition of phosphorus-cycle genes in rice field soil. Nevertheless, the presence of these diverse P-cycle genes can enhance the dissolution of phosphate rocks or maintain P solubility in the soil. Interestingly, the application of 90 kg P_2_O_5_ ha^−1^ only in the first year of cultivation did not affect the composition of soil P-cycle genes, in contrast to findings by Liu et al. (2017) and (2022), who observed modifications in the composition of inorganic P-solubilization and organic P-mineralization genes in soil due to long-term high-P inputs (200 kg P_2_O_5_ ha^−1^ year^−1^).

Furthermore, regardless of the applied treatment, the phoD and ppk genes, are the significantly most abundant genes in these soils. The abundance of these P-cycle genes indicates that microorganisms in the rice field soils of the Man research station have a strong potential to mineralize organic phosphates in the soil through alkaline phosphatases (phoD) and acid phosphatases (appA, 3-phytases) (Li et al., 2018; Dai et al., 2020) and to solubilize inorganic phosphates through quinoprotein glucose dehydrogenases (gcd) and polyphosphate kinases (ppk) (Siles et al., 2022).

However, our findings revealed that the application of soluble NPK alone did not alter the composition of phosphorus cycle genes in the studied soils but modified their abundance by decreasing the abundance of inorganic P-solubilization (ppk and gcd) and organic P-mineralization genes (3-phytase, appA, and phoD).

Moreover, the input of natural phosphate rock from Morocco (100% PRM) as phosphate amendment significantly reduces the abundance of appA and gcd genes, have various effect on the abundance of ppK genes, according on soil characteristic, and have no significant effect on 3-phytase and phoD genes compared to the unamended plot (T0a).

The exclusive use of Triple Super Phosphate (TSP) as the phosphate amendment resulted in notable changes in the abundance of phosphorus genes. Specifically, there was a significant decrease in the abundance of ppk, gcd, appA, and phoD genes. In contrast, the abundance of 3-phytase showed no significant effect, exhibiting both increases and decreases in abundance of ppk, phoD, and 3-phytase genes, depending on the soil characteristic (Liu et al., 2023).

However, when plots were amended with a combination of phosphate rock and triple superphosphate (TSP), there was a reduction in the abundance of appA, 3-phytase, and gcd genes and an enhancement in the abundance of 3-phytase and ppk genes, alongside varying effects of PRM on the abundance of phoD depending on soil characteristic.

It appears that the nature of phosphorus sources does not alter the composition of phosphorus cycle genes but significantly modifies the abundance of P-cycle genes, depending on soil characteristics or environmental factors. This variation is attributed to the nature of the amendment and also to environment factors.

## Conclusion

The analysis of bacterial diversity in paddy field soils reveals the presence of various bacterial groups, including the Firmicutes phylum, which are known for their ability to solubilize and mineralize organic and inorganic phosphates. Additionally, the identification of five different genes involved in the phosphorus cycle could contribute to the effective dissolution of applied phosphate amendments. This study highlights variable changes in the abundance of bacterial communities and phosphorus cycle genes depending on both the phosphorus source in the amendments and environmental factors. However, combining natural phosphate rock with chemical fertilizers appears to mitigate this impact on soil microorganism abundance.

## Data availability statement

The original contributions presented in the study are publicly available. This data can be found here: Github (https://github.com/BONGOUA/BONGOUA-DEVISME--10.3389-fmicb.2024.1409559/commit/9f0f392b9b9dbc92d33b6f55c0630e406e9eee1f) and NCBI BioSample (https://www.ncbi.nlm.nih.gov/biosample/), accession numbers SAMN43473894- SAMN43473925.

## Author contributions

AB-D: Formal analysis, Supervision, Writing – review & editing, Writing – original draft. SK: Methodology, Writing – original draft. K-KK: Supervision, Writing – review & editing. BL: Methodology, Writing – review & editing.
